# Mannose Treatment: A Promising Novel Strategy to Suppress Inflammation

**DOI:** 10.3389/fimmu.2021.756920

**Published:** 2021-09-27

**Authors:** Wei Zhang, Hao Cheng, Yuanyuan Gui, Qipeng Zhan, Si Li, Wenliang Qiao, Aiping Tong

**Affiliations:** ^1^ State Key Laboratory of Biotherapy and Cancer Center, West China Hospital, Sichuan University, Chengdu, China; ^2^ Discovery Project Unit, HitGen Inc. Tianfu International Bio-Town, Chengdu, China

**Keywords:** mannose, inflammation, hexose, inflammatory diseases, mannose treatment

## Abstract

High glucose and fructose intake have been proven to display pro-inflammatory roles during the progression of inflammatory diseases. However, mannose has been shown to be a special type of hexose that has immune regulatory functions. In this review, we trace the discovery process of the regulatory functions of mannose and summarize some past and recent studies showing the therapeutic functions of mannose in inflammatory diseases. We conclude that treatment with mannose can suppress inflammation by inducing regulatory T cells, suppressing effector T cells and inflammatory macrophages, and increasing anti-inflammatory gut microbiome. By summarizing all the important findings, we highlight that mannose treatment is a safe and promising novel strategy to suppress inflammatory diseases, including autoimmune disease and allergic disease.

## Introduction

Sugar intake, mainly glucose and fructose, within generic diets has increased dramatically during the past century. Hexose, especially glucose, is the most important energy source in living organisms. However, it has been well documented that consuming too much sugar can raise the incidence of many health problems, including diabetes and obesity. Recently, more studies have demonstrated the harmful effects of sugar. For example, one study reported that glucose-fructose syrup (HFCS) enhances intestinal tumor growth in mice *via* activation of glycolysis and increased synthesis of fatty acids in tumor cells that support tumor growth (1); Another study found that high fructose intake is associated with increased hepatic fatty acid synthesis and marked insulin resistance (2), and a final study showed that dietary fructose feeds hepatic lipogenesis *via* microbiota-derived acetate (3). More than that, one clinical study among US adults found that consumption of sugar-sweetened beverages (SSBs) is positively associated with total mortality (4).

In addition, high glucose intake and high fructose intake have also been proven to have pro-inflammatory roles for inflammatory diseases. Zhang et al. revealed that high glucose intake exacerbates autoimmunity in mouse models of colitis and experimental autoimmune encephalomyelitis (EAE) by promoting T helper-17 (Th17) cell differentiation (5). Jones et al. found that fructose reprograms glutamine-dependent oxidative metabolism in mononuclear phagocytes to support LPS-induced inflammation (6). Another study found that high-fructose diet (HFrD) elicited endotoxemia, could activate toll-like receptor (TLR) signaling in liver macrophages, and induce liver inflammation (7). Another two studies also reported that dietary simple sugars alter microbial ecology in the gut and promote colitis and EAE in mice (8, 9).

Surprisingly, not all hexoses are pathogenic or pro-inflammatory. During the past few years, the immune regulatory functions of mannose, a C-2 epimer of glucose, have been revealed (10). Although mannose has been shown to be effective in the treatment of bacterial urinary tract infections by blocking the adhesion of enteric bacteria to uroepithelial cells (11–14), it was thought for a long time that the key function of mannose was to glycosylate certain proteins (15, 16), and that a mannose supplement must be given to the individuals with congenital disorders of glycosylation type Ib to support their survival (17, 18). Recently, quite a few studies have highlighted the fact that mannose is an effective suppressor of inflammation and autoimmunity (10, 19–21). Mannose has been shown to suppress numerous inflammatory diseases, including Type I diabetes (T1D) (10), asthma (10), colitis (19), obesity (20), osteoarthritis (22), chronic graft-versus- host disease (cGVHD) and lupus (21). In this review, we summarize the mechanisms of immunomodulatory effects mediated by mannose treatment, highlight mannose treatment as a promising strategy to suppress inflammation, and point to the remaining key questions that need to be addressed urgently in further studies.

## Regulatory Functions of Mannose in Inflammation

### Mannose Induces Regulatory T Cells and Suppresses Effector T Cells

**Figure 1:**
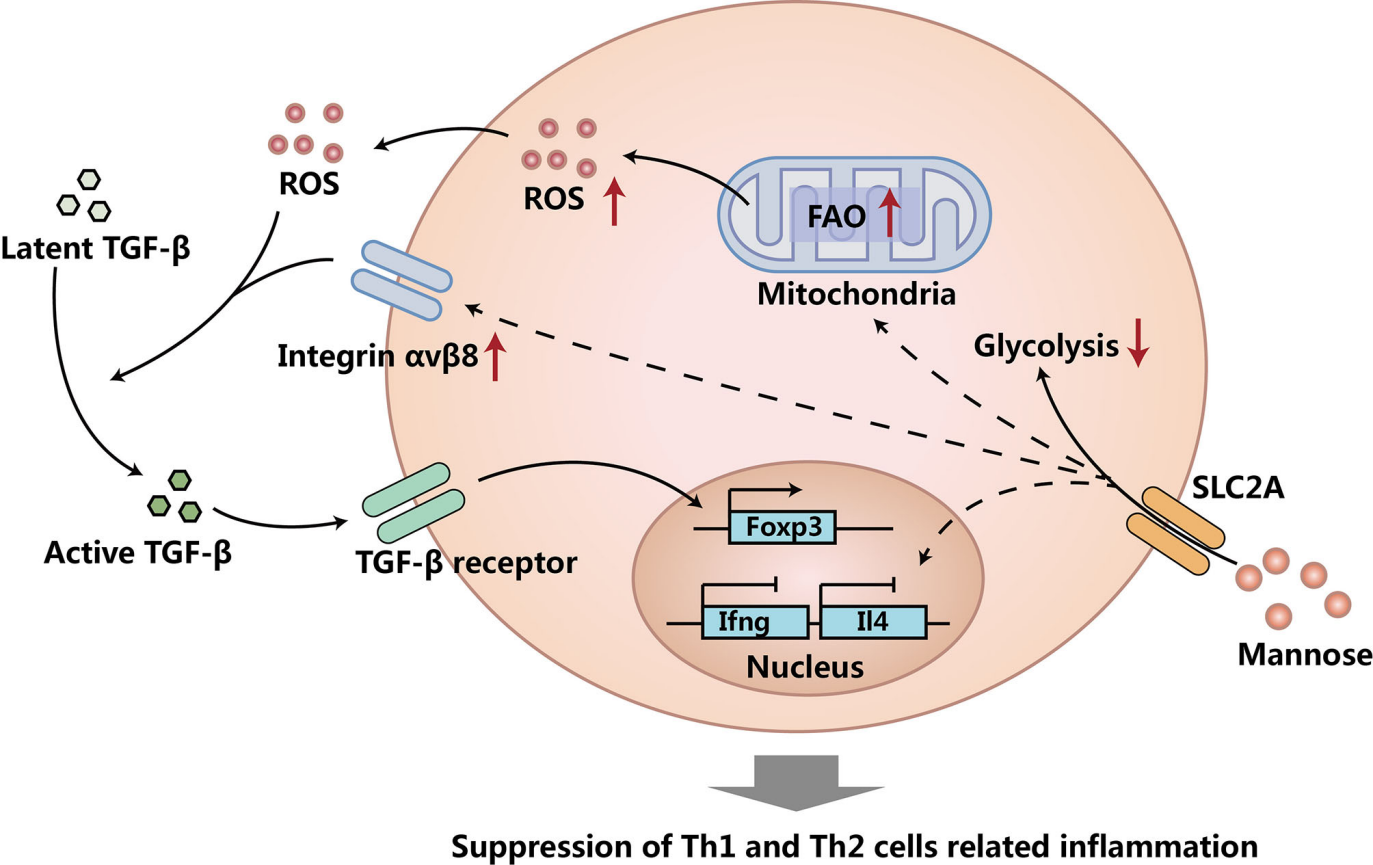
Mannose induces Treg cells and suppresses Teff cells. Mannose suppresses glycolysis, increases fatty acid oxidation (FAO), and up-regulates integrin α_v_β_8_ in CD4^+^ T cells. Increased FAO caused more reactive oxygen species (ROS) production. The increased ROS and up-regulated integrin α_v_β_8_ activates more transforming growth factor beta (TGF-β) from its latent form. TGF-β induces more Treg cells, suppresses Th1 and Th2 cells, and causes the suppression of Th1 and Th2 cells related to inflammation. Mechanisms of Treg cell and TGF-β independent Th1 and Th2 cell suppression caused by mannose treatment needs to be further investigated.

CD4^+^CD25^+^Foxp3^+^ regulatory T cells (Treg cells) are the most important cell population to maintain tolerance to self-antigens (23) and harmless antigens (24) (e.g. pollen) by suppressing responses of effector T cells (Teff cells) and other immune cell responses (25–28). Interestingly, Zhang et al. reported that supraphysiological levels of mannose supplemented orally through drinking water could induce Treg cells and suppress Type I diabetes and OVA-induced airway inflammation in mouse models (10). They found that mannose induced Treg cells both *in vivo* and *in vitro* through the activation of transforming growth factor beta (TGF-β) from its latent form, and they further found that the activation of TGF-β induced by mannose was mediated by increased reactive oxygen species (ROS) and integrin α_v_β_8_. Interestingly, the increased ROS production induced by mannose was from fatty acid oxidation (FAO), as mannose treatment increased FAO, but suppressed aerobic glycolysis in CD4^+^ T cells. Moreover, mannose could also suppress type 1 helper T cells (Th1 cells) and type 2 helper T cells (Th2 cells) in a Treg cell and TGF-β independent mechanism, as they found that mannose could suppress Th1 cell cytokines (*Ifng* and *Il2*) and Th2 cell cytokines (*Il4* and *Il13*) during CD4^+^ T cell activation (before the generation of Treg cells), and they also showed that Treg cell depletion *in vivo* did not increase Th1 cell frequency in NOD mice treated with mannose (10). Overall, this encompassing work revealed the immune regulatory functions of mannose by inducing Treg cells and suppressing Teff cells, and provided a fascinating new insight into the beneficial effects of this unique sugar (**Figure 1**) **(** 10). These findings suggested that mannose could be a safe dietary supplement to promote immune tolerance and to treat/prevent human diseases associated with autoimmunity and allergy (29). In the meantime, mannose treatment did not affect *Il17* expression, suggesting that mannose may specifically induce Treg cells without affecting other protective responses such as Th17 cell-mediated gut integrity (30).

Although the clear mechanism of mannose induced Th1 and Th2 cell suppression is still unknown, it is very likely that the possible mechanism of the Treg cell and TGF-β independent suppression of Th1 cells and Th2 cells might be through the suppression of aerobic glycolysis in CD4^+^ T cells, as it has been well-proven that both differentiations and functions of Th1 cells and Th2 cells largely rely on aerobic glycolysis (31, 32). Besides suppressing Th1 and Th2 cells *via* a TGF-β activation independent mechanism, mannose might also have other mechanisms to promote Treg cell generation. One study reported that mannose treated mesenchymal stem cells (MSCs) could induce more Treg cells *via* the suppression of Interleukin 6 (IL-6) produced by MSCs (33). All these findings show that mannose can suppress inflammation *via* induction of Treg cells and suppression of Teff cells.

### Mannose Suppresses Macrophage-Mediated Inflammation

**Figure 2:**
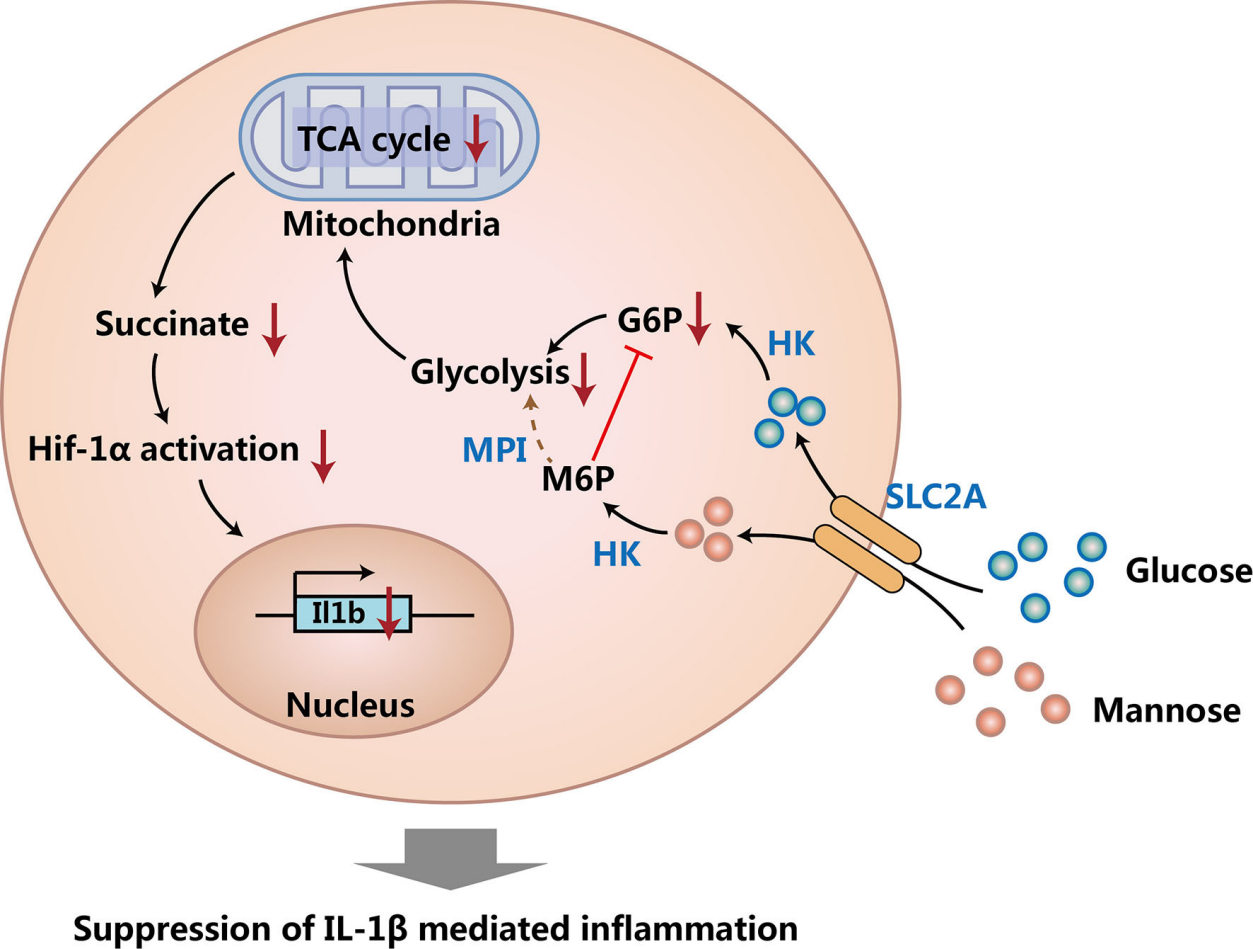
Mannose suppresses macrophage IL-1β production. Mannose and glucose share the same transporter (SLC2A) and can be converted to mannose 6-phosphate (M6P) and glucose 6-phosphate (G6P) respectively by hexokinase (HK). The process of M6P generation suppresses glycolysis by reducing G6P, and M6P cannot be used for glycolysis efficiently due to the low expression of phosphomannose isomerase (MPI) in macrophages. Suppressed glycolysis causes reduced tricarboxylic acid (TCA) cycle and decreases the production of succinate. Decreased succinate reduces succinate-mediated HIF-1α activation, and then causes the decreased expression of IL-1β in macrophages.

Macrophages are a critical immune cell population that are important for innate immunity (34). Besides suppressing Th1 cells and Th2 cells, mannose has also been found to suppress inflammatory macrophages (19). First of all, Torretta et al. proved that mannose could limit the production of Interleukin 1β (IL-1β) and suppress the activation of lipopolysaccharide (LPS)-induced macrophages *in vitro*. On the other hand, mannose could also promote the survival of LPS-treated mice *in vivo*. Next, they found that mannose suppressed macrophage-derived IL-1β production by reducing glycolysis, tricarboxylic acid (TCA) cycle, and suppressing succinate-mediated HIF-1α activation in macrophages. Since mannose cannot be used for glycolysis efficiently, these findings further showed that mannose reduced glycolysis by competing glucose transporter (SLC2A, also called GLUT) and hexokinase (HK) with glucose, and the reduced glycolysis caused the reduction of TCA cycle. Moreover, they also demonstrated that mannose could suppress dextran sulfate sodium (DSS) induced colitis in mice *via* limiting glycolysis, TCA cycle, and IL-1β production in macrophages. Taken together, this fantastic work revealed the immune regulatory function of mannose by suppressing IL-1β production of inflammatory macrophages (**Figure 2**) **(** 19).

Interestingly, the suppression of macrophage IL-1β production completely relies on the low expression of phosphomannose isomerase (MPI) in macrophages, as overexpression of MPI in macrophages can overcome the suppression function of mannose (19). Since MPI could convert mannose-6-phosphate to fructose-6-phophate and then use fructose-6-phophate for glycolysis (34), these findings suggest that mannose can suppress glycolysis in cells expressing low amounts of MPI by competing hexokinase (HK) with glucose; whereas in cells expressing high amounts of MPI, mannose-6-phosphate could be converted to fructose-6-phophate efficiently by MPI, therefore mannose could support glycolysis quite efficiently in these cells. Since it was found that mannose could suppress glycolysis in CD4+ T cells (10), we can presume that the expression of MPI in CD4^+^ T cells should also be quite low. Besides suppressing macrophage IL-1β production, one study found that mannose treatment promoted proliferation, enhanced autophagy, and reduced apoptosis of IL-1β-treated rat chondrocytes, and therefore suppressed the progression of osteoarthritis (OA) (22). All of these findings show that mannose not only suppresses IL-1β production of macrophages, but also protects bodies from IL-1β induced degeneration.

### Mannose May Increase Anti-Inflammatory Gut Microbiome

Within the past decade, the gut microbiome has been proven to have critical functions in immune homeostasis and inflammation (35, 36). Sharma et al. showed that mannose treatment by drinking-water supplementation prevented weight gain, lowered adiposity, reduced liver steatosis, and improved glucose tolerance during the induction of obesity in high-fat diet (HFD) mice (20). Interestingly, these beneficial effects of mannose were observed only when initiated early in life, but not when provided later. These changes in HFD mice with different ages, coupled with the fact that continuous mannose supplementation is required, made authors postulate that mannose treatment might change gut microbiome in HFD mice. Indeed, they proved that mannose treatment initiated early in life increased the *Bacteroidetes* to *Firmicutes* ratio in the gut microbiota of HFD mice, showing an association between gut microbiota composition and the timing of mannose introduction (20). The decrease of the *Bacteroidetes* to *Firmicutes* ratio in the gut microbiota has been shown to cause obesity in both mice and human, and the increase of the ratio is positively associated with weight loss (37–39). These findings show that mannose suppressed obesity by increasing the *Bacteroidetes* to *Firmicutes* ratio in the gut microbiota.

Importantly, *Bacteroides* are the most abundant members of *Bacteroidetes* in the intestinal tract of mammals, and the immunomodulatory activities of *Bacteroides* have been identified (40–43). Lower levels of *Bacteroides* in the gut microbiota have been shown to be associated with Inflammatory Bowel Disease (IBD) (40), and polysaccharide A expressed by *Bacteroides* can induce Treg cell growth and suppress immunity (42). Consistent with these findings, mannose treatment increased *Bacteroidetes* and also reduced gene expression of inflammatory markers (*Tnfa* and *Ifng*) in adipocytes of HFD mice (20). Another study also reported that mannose treatment attenuated bone loss induced by senility and estrogen deficiency in mice, *via* the increase of Treg cells and *Bacteroidetes (* 44). These findings suggest mannose treatment has gut microbiota-dependent anti-inflammatory effects.

However, an increase in *Bacteroides* is not necessarily always beneficial for anti-inflammation (45). For example, one comparative study reported that *Bacteroides vulgatus* (a genera of *Bacteroides*) in the outer membrane might be implicated in the pathogenesis of ulcerative colitis (46), another study found the growth inhibition of the *Bacteroides vulgatus* may repress exacerbation of intestinal inflammation (47). So mannose associated increases in *Bacteroidetes* might not necessarily be the main mechanism in which chronic inflammation is suppressed. Taken together, mannose treatment may have gut microbiota-dependent anti-inflammatory effects, but the clear mechanisms need to be investigated further.

## Conclusion and Future Perspective

High sugar intake has long been shown to have pathogenic roles in a variety of diseases, including diabetes and obesity. Recently, functions of high-sugar intake in autoimmunity were also revealed (5, 8, 48). Unlike glucose and fructose, mannose is a special hexose that suppresses inflammation (10, 49). Also, the therapeutic concentration of circulating mannose can be reached in mice and humans (50, 51), and even a very low concentration of mannose supplemented in drinking water has a therapeutic function both *in vitro* and *in vivo* (10, 20). More than that, mannose treatment is supposed to be a safe treatment, as long-term mannose ingestion was well tolerated and did not show any adverse effect in mice and humans (50, 51).

On the other hand, there are still many important questions that need to be answered in the future. For example, it is necessary to calculate the ideal dose of mannose treatment for human inflammation, determine how mannose increases FAO in CD4+ T cells, and whether or not mannose can affect functions of CD8^+^ T cell and other immune cells. Moreover, since it was also reported that mannose treatment could enhance cancer chemokine therapy (52, 53), whether mannose mediated immune responses are involved during this process is totally unknown. Nevertheless, although there are still a number of key questions need to be figured out, the great therapeutic promise of mannose treatment has been disclosed.

In conclusion, mannose treatment is a promising novel strategy to suppress inflammatory diseases, including autoimmune disease and allergic disease. More intense study and research would greatly benefit patients with inflammatory disease.

## Author Contributions

WZ and HC wrote the manuscript. YG, QZ, SL, and WQ edited the manuscript. AT designed the layout of manuscript and edited the manuscript. All authors contributed to the article and approved the submitted version.

## Funding

This work was supported by the National Natural Science Foundation of China (NO. 82073404), and Major Subject of the Science and Technology Department of Sichuan Province (2020YFS0251).

## Conflict of Interest

Author SL is employed by HitGen Inc.

The remaining authors declare that the research was conducted in the absence of any commercial or financial relationships that could be construed as a potential conflict of interest.

## Publisher’s Note

All claims expressed in this article are solely those of the authors and do not necessarily represent those of their affiliated organizations, or those of the publisher, the editors and the reviewers. Any product that may be evaluated in this article, or claim that may be made by its manufacturer, is not guaranteed or endorsed by the publisher.

## References

[1] Goncalves MD Lu C Tutnauer J Hartman TE Hwang SK Murphy CJ. High-Fructose Corn Syrup Enhances Intestinal Tumor Growth in Mice. Science (2019) 363(6433):1345–9. doi: 10.1126/science.aat8515 PMC6487857 30898933

[2] Softic S Gupta MK Wang GX Fujisaka S O’Neill BT Rao TN. Divergent Effects of Glucose and Fructose on Hepatic Lipogenesis and Insulin Signaling. J Clin Invest (2017) 127(11):4059–74. doi: 10.1172/JCI94585 PMC5663363 28972537

[3] Zhao S Jang C Liu J Uehara K Gilbert M Izzo L. Dietary Fructose Feeds Hepatic Lipogenesis *via* Microbiota-Derived Acetate. Nature (2020) 579(7800):586–91. doi: 10.1038/s41586-020-2101-7 PMC7416516 32214246

[4] Malik VS Li Y Pan A De Koning L Schernhammer E Willett WC. Long-Term Consumption of Sugar-Sweetened and Artificially Sweetened Beverages and Risk of Mortality in US Adults. Circulation (2019) 139(18):2113–25. doi: 10.1161/CIRCULATIONAHA.118.037401 PMC6488380 30882235

[5] Zhang D Jin W Wu R Li J Park SA Tu E. High Glucose Intake Exacerbates Autoimmunity Through Reactive-Oxygen-Species-Mediated TGF-Beta Cytokine Activation. Immunity (2019) 51(4):671–81.e5. doi: 10.1016/j.immuni.2019.08.001 31451397 PMC9811990

[6] Jones N Blagih J Zani F Rees A Hill DG Jenkins BJ. Fructose Reprogrammes Glutamine-Dependent Oxidative Metabolism to Support LPS-Induced Inflammation. Nat Commun (2021) 12(1):1209. doi: 10.1038/s41467-021-21461-4 33619282 PMC7900179

[7] Todoric J Di Caro G Reibe S Henstridge DC Green CR Vrbanac A. Fructose Stimulated *De Novo* Lipogenesis Is Promoted by Inflammation. Nat Metab (2020) 2(10):1034–45. doi: 10.1038/s42255-020-0261-2 PMC8018782 32839596

[8] Khan S Waliullah S Godfrey V Khan MAW Ramachandran RA Cantarel BL. Dietary Simple Sugars Alter Microbial Ecology in the Gut and Promote Colitis in Mice. Sci Transl Med (2020) 12(567):eaay6218. doi: 10.1126/scitranslmed.aay6218 33115951

[9] Cao G Wang Q Huang W Tong J Ye D He Y. Long-Term Consumption of Caffeine-Free High Sucrose Cola Beverages Aggravates the Pathogenesis of EAE in Mice. Cell Discov (2017) 3:17020. doi: 10.1038/celldisc.2017.20 28670480 PMC5477007

[10] Zhang D Chia C Jiao X Jin W Kasagi S Wu R. D-Mannose Induces Regulatory T Cells and Suppresses Immunopathology. Nat Med (2017) 23(9):1036–45. doi: 10.1038/nm.4375 PMC12180587 28759052

[11] Michaels EK Chmiel JS Plotkin BJ Schaeffer AJ. Effect of D-Mannose and D-Glucose on Escherichia Coli Bacteriuria in Rats. Urol Res (1983) 11(2):97–102. doi: 10.1007/BF00256954 6346629

[12] Schaeffer AJ Chmiel JS Duncan JL Falkowski WS. Mannose-Sensitive Adherence of Escherichia Coli to Epithelial Cells From Women With Recurrent Urinary Tract Infections. J Urol (1984) 131(5):906–10. doi: 10.1016/s0022-5347(17)50706-5 6142969

[13] Kranjcec B Papes D Altarac S. D-Mannose Powder for Prophylaxis of Recurrent Urinary Tract Infections in Women: A Randomized Clinical Trial. World J Urol (2014) 32(1):79–84. doi: 10.1007/s00345-013-1091-6 23633128

[14] Lenger SM Bradley MS Thomas DA Bertolet MH Lowder JL. Sutcliffe S. D-Mannose *vs* Other Agents for Recurrent Urinary Tract Infection Prevention in Adult Women: A Systematic Review and Meta-Analysis. Am J Obstet Gynecol (2020) 223(2):265.e1–13. doi: 10.1016/j.ajog.2020.05.048 PMC7395894 32497610

[15] Alton G Hasilik M Niehues R Panneerselvam K Etchison JR Fana F. Direct Utilization of Mannose for Mammalian Glycoprotein Biosynthesis. Glycobiology (1998) 8(3):285–95. doi: 10.1093/glycob/8.3.285 9451038

[16] Freeze HH Sharma V. Metabolic Manipulation of Glycosylation Disorders in Humans and Animal Models. Semin Cell Dev Biol (2010) 21(6):655–62. doi: 10.1016/j.semcdb.2010.03.011 PMC2917643 20363348

[17] Schneider A Thiel C Rindermann J DeRossi C Popovici D Hoffmann GF. Successful Prenatal Mannose Treatment for Congenital Disorder of Glycosylation-Ia in Mice. Nat Med (2011) 18(1):71–3. doi: 10.1038/nm.2548 22157680

[18] de Lonlay P Seta N. The Clinical Spectrum of Phosphomannose Isomerase Deficiency, With an Evaluation of Mannose Treatment for CDG-Ib. Biochim Biophys Acta (2009) 1792(9):841–3. doi: 10.1016/j.bbadis.2008.11.012 19101627

[19] Torretta S Scagliola A Ricci L Mainini F Di Marco S Cuccovillo I. D-Mannose Suppresses Macrophage IL-1beta Production. Nat Commun (2020) 11(1):6343. doi: 10.1038/s41467-020-20164-6 33311467 PMC7733482

[20] Sharma V Smolin J Nayak J Ayala JE Scott DA Peterson SN. Mannose Alters Gut Microbiome, Prevents Diet-Induced Obesity, and Improves Host Metabolism. Cell Rep (2018) 24(12):3087–98. doi: 10.1016/j.celrep.2018.08.064 PMC6207501 30231992

[21] Wang H Teng X Abboud G Li W Ye S. Morel L. D-Mannose Ameliorates Autoimmune Phenotypes in Mouse Models of Lupus. BMC Immunol (2021) 22(1):1. doi: 10.1186/s12865-020-00392-7 33402096 PMC7786459

[22] Lin Z Miao J Zhang T He M Zhou X Zhang H. D-Mannose Suppresses Osteoarthritis Development *In Vivo* and Delays IL-1beta-Induced Degeneration *In Vitro* by Enhancing Autophagy Activated *via* the AMPK Pathway. BioMed Pharmacother (2021) 135:111199. doi: 10.1016/j.biopha.2020.111199 33401221

[23] Zhang D Tu E Kasagi S Zanvit P Chen Q Chen W. Manipulating Regulatory T Cells: A Promising Strategy to Treat Autoimmunity. Immunotherapy (2015) 7(11):1201–11. doi: 10.2217/imt.15.79 PMC4976828 26568117

[24] Akdis M Blaser K Akdis CA. T Regulatory Cells in Allergy: Novel Concepts in the Pathogenesis, Prevention, and Treatment of Allergic Diseases. J Allergy Clin Immunol (2005) 116(5):961–8; quiz 969. doi: 10.1016/j.jaci.2005.09.004 16275361

[25] Sakaguchi S Sakaguchi N Asano M Itoh M Toda M. Immunologic Self-Tolerance Maintained by Activated T Cells Expressing IL-2 Receptor Alpha-Chains (CD25). Breakdown of a Single Mechanism of Self-Tolerance Causes Various Autoimmune Diseases. J Immunol (1995) 155(3):1151–64. 7636184

[26] Hori S Nomura T Sakaguchi S. Control of Regulatory T Cell Development by the Transcription Factor Foxp3. Science (2003) 299(5609):1057–61. doi: 10.1126/science.1079490 12522256

[27] Fontenot JD Gavin MA Rudensky AY. Foxp3 Programs the Development and Function of CD4+CD25+ Regulatory T Cells. Nat Immunol (2003) 4(4):330–6. doi: 10.1038/ni904 12612578

[28] Khattri R Cox T Yasayko SA Ramsdell F. An Essential Role for Scurfin in CD4+CD25+ T Regulatory Cells. Nat Immunol (2003) 4(4):337–42. doi: 10.1038/ni909 12612581

[29] Shi YB Yin D. A Good Sugar, D-Mannose, Suppresses Autoimmune Diabetes. Cell Biosci (2017) 7:48. doi: 10.1186/s13578-017-0175-1 29021891 PMC5613377

[30] Villa M Qiu J. Pearce EL. A Sweet Deal for Diabetes. Trends Endocrinol Metab (2018) 29(1):1–2. doi: 10.1016/j.tem.2017.10.006 29102469

[31] Michalek RD Gerriets VA Jacobs SR Macintyre AN MacIver NJ Mason EF. Cutting Edge: Distinct Glycolytic and Lipid Oxidative Metabolic Programs Are Essential for Effector and Regulatory CD4+ T Cell Subsets. J Immunol (2011) 186(6):3299–303. doi: 10.4049/jimmunol.1003613 PMC3198034 21317389

[32] MacIver NJ Michalek RD Rathmell JC. Metabolic Regulation of T Lymphocytes. Annu Rev Immunol (2013) 31:259–83. doi: 10.1146/annurev-immunol-032712-095956 PMC3606674 23298210

[33] Guo L Hou Y Song L Zhu S Lin F. Bai Y. D-Mannose Enhanced Immunomodulation of Periodontal Ligament Stem Cells *via* Inhibiting IL-6 Secretion. Stem Cells Int (2018) 2018:7168231. doi: 10.1155/2018/7168231 30271438 PMC6151224

[34] Sharma V Ichikawa M Freeze HH. Mannose Metabolism: More Than Meets the Eye. Biochem Biophys Res Commun (2014) 453(2):220–8. doi: 10.1016/j.bbrc.2014.06.021 PMC4252654 24931670

[35] Belkaid Y Hand TW. Role of the Microbiota in Immunity and Inflammation. Cell (2014) 157(1):121–41. doi: 10.1016/j.cell.2014.03.011 PMC4056765 24679531

[36] Zheng D Liwinski T Elinav E. Interaction Between Microbiota and Immunity in Health and Disease. Cell Res (2020) 30(6):492–506. doi: 10.1038/s41422-020-0332-7 32433595 PMC7264227

[37] Ley RE Backhed F Turnbaugh P Lozupone CA Knight RD Gordon JI. Obesity Alters Gut Microbial Ecology. Proc Natl Acad Sci USA (2005) 102(31):11070–5. doi: 10.1073/pnas.0504978102 PMC1176910 16033867

[38] Ley RE Turnbaugh PJ Klein S Gordon JI. Microbial Ecology: Human Gut Microbes Associated With Obesity. Nature (2006) 444(7122):1022–3. doi: 10.1038/4441022a 17183309

[39] Ridaura VK Faith JJ Rey FE Cheng J Duncan AE Kau AL. Gut Microbiota From Twins Discordant for Obesity Modulate Metabolism in Mice. Science (2013) 341(6150):1241214. doi: 10.1126/science.1241214 24009397 PMC3829625

[40] Zhou Y Zhi F. Lower Level of Bacteroides in the Gut Microbiota Is Associated With Inflammatory Bowel Disease: A Meta-Analysis. BioMed Res Int (2016) 2016:5828959. doi: 10.1155/2016/5828959 27999802 PMC5143693

[41] Mazmanian SK Round JL Kasper DL. A Microbial Symbiosis Factor Prevents Intestinal Inflammatory Disease. Nature (2008) 453(7195):620–5. doi: 10.1038/nature07008 18509436

[42] Round JL Lee SM Li J Tran G Jabri B Chatila TA. The Toll-Like Receptor 2 Pathway Establishes Colonization by a Commensal of the Human Microbiota. Science (2011) 332(6032):974–7. doi: 10.1126/science.1206095 PMC3164325 21512004

[43] Delday M Mulder I Logan ET Grant G. Bacteroides Thetaiotaomicron Ameliorates Colon Inflammation in Preclinical Models of Crohn’s Disease. Inflammation Bowel Dis (2019) 25(1):85–96. doi: 10.1093/ibd/izy281 PMC6290787 30215718

[44] Liu H Gu R Zhu Y Lian X Wang S Liu X. D-Mannose Attenuates Bone Loss in Mice *via* Treg Cell Proliferation and Gut Microbiota-Dependent Anti-Inflammatory Effects. Ther Adv Chronic Dis (2020) 11:2040622320912661. doi: 10.1177/2040622320912661 32341776 PMC7169364

[45] Zhang SL Wang SN Miao CY. Influence of Microbiota on Intestinal Immune System in Ulcerative Colitis and Its Intervention. Front Immunol (2017) 8:1674. doi: 10.3389/fimmu.2017.01674 29234327 PMC5712343

[46] Bamba T Matsuda H Endo M Fujiyama Y. The Pathogenic Role of Bacteroides Vulgatus in Patients With Ulcerative Colitis. J Gastroenterol (1995) 30 Suppl 8:45–7. 8563888

[47] Setoyama H Imaoka A Ishikawa H Umesaki Y. Prevention of Gut Inflammation by Bifidobacterium in Dextran Sulfate-Treated Gnotobiotic Mice Associated With Bacteroides Strains Isolated From Ulcerative Colitis Patients. Microbes Infect (2003) 5(2):115–22. doi: 10.1016/s1286-4579(02)00080-1 12650769

[48] Galgani M Matarese G. The Sweet Kiss Breaching Immunological Self-Tolerance. Trends Mol Med (2019) 25(10):819–20. doi: 10.1016/j.molmed.2019.08.003 31451384

[49] Wei Z Huang L Cui L Zhu X. Mannose: Good Player and Assister in Pharmacotherapy. BioMed Pharmacother (2020) 129:110420. doi: 10.1016/j.biopha.2020.110420 32563989

[50] Mayatepek E Schroder M Kohlmuller D Bieger WP Nutzenadel W. Continuous Mannose Infusion in Carbohydrate-Deficient Glycoprotein Syndrome Type I. Acta Paediatr (1997) 86(10):1138–40. doi: 10.1111/j.1651-2227.1997.tb14825.x 9350901

[51] Davis JA Freeze HH. Studies of Mannose Metabolism and Effects of Long-Term Mannose Ingestion in the Mouse. Biochim Biophys Acta (2001) 1528(2-3):116–26. doi: 10.1016/s0304-4165(01)00183-0 11687298

[52] Gonzalez PS O’Prey J Cardaci S Barthet VJA Sakamaki JI Beaumatin F. Mannose Impairs Tumour Growth and Enhances Chemotherapy. Nature (2018) 563(7733):719–23. doi: 10.1038/s41586-018-0729-3 30464341

[53] Liu F Xu X Li C Li C Li Y Yin S. Mannose Synergizes With Chemoradiotherapy to Cure Cancer *via* Metabolically Targeting HIF-1 in a Novel Triple-Negative Glioblastoma Mouse Model. Clin Transl Med (2020) 10(7):e226. doi: 10.1002/ctm2.226 33252849 PMC7648968

